# The Race of CAR Therapies: CAR-NK Cells for Fighting B-Cell Hematological Cancers

**DOI:** 10.3390/cancers13215418

**Published:** 2021-10-28

**Authors:** Lara Herrera, Silvia Santos, Miguel Angel Vesga, Tomas Carrascosa, Juan Carlos Garcia-Ruiz, Antonio Pérez-Martínez, Manel Juan, Cristina Eguizabal

**Affiliations:** 1 Cell Therapy, Stem Cells and Tissues Group, Biocruces Bizkaia Health Research Institute, 48903 Barakaldo, Bizkaia, Spain; lara.herreradelval@osakidetza.eus (L.H.); silvia.santoscabrera@osakidetza.eus (S.S.); miguelangel.vesgacarasa@osakidetza.eus (M.A.V.); 2 Basque Centre for Blood Transfusion and Human Tissues, 48960 Galdakao, Bizkaia, Spain; 3 Hematology and Hemotherapy Service, Galdakao-Usansolo University Hospital, 48960 Galdakao, Bizkaia, Spain; tomas.carrascosavallejo@osakidetza.eus; 4 Hematological Cancer Group, Biocruces Bizkaia Health Research Institute, 48903 Barakaldo, Bizkaia, Spain; juancarlos.garciaruiz@osakidetza.eus; 5 Hematology and Hemotherapy Service, Cruces University Hospital, 48903 Barakaldo, Bizkaia, Spain; 6 Faculty of Medicine and Nursing, University of the Basque Country UPV/EHU, 48940 Leioa, Bizkaia, Spain; 7 Translational Research in Pediatric Oncology, Hematopoietic Transplantation and Cell Therapy, Hospital La Paz Institute for Health Research (IdiPAZ), 28046 Madrid, Spain; aperezmartinez@salud.madrid.org; 8 Faculty of Medicine, Universidad Autónoma de Madrid, 28046 Madrid, Spain; 9 Pediatric Hemato-Oncology Department, Hospital Universitario La Paz, 28046 Madrid, Spain; 10 Clinical Immunology Program, Hospital Sant Joan de Déu-Hospital Clínic Barcelona, 08950 Esplugues de Llobregat, Barcelona, Spain; mjuan@clinic.cat; 11 Immunotherapy Platform, Hospital Sant Joan de Déu-Hospital Clínic, Universitat de Barcelona, 08950 Esplugues de Llobregat, Barcelona, Spain; 12 Department of Immunology-CDB, Hospital Clínic-IDIBAPS, 08007 Barcelona, Spain; 13 Universitat de Barcelona, 08007 Barcelona, Spain

**Keywords:** CAR-NK, CAR-T, hematological cancer, immunotherapy, blood

## Abstract

**Simple Summary:**

Over the last few years, CAR-T cells have arisen as one of the most promising immunotherapies against relapsed or refractory hematological cancers. Despite their good results in clinical trials, there are some limitations to overcome, such as undesirable side-effects or the restraints of an autologous treatment. Therefore, CAR-NK cells have emerged as a good alternative for these kinds of treatments. This review discusses the advantages of CAR-NK cells compared to CAR-T cells, as well as the different sources and strategies in order to obtain these CAR-NK cells.

**Abstract:**

Acute lymphoblastic leukemia (ALL) and Chronic lymphocytic leukemia (CLL) are the most common leukemias in children and elderly people, respectively. Standard therapies, such as chemotherapy, are only effective in 40% of ALL adult patients with a five-year survival rate and therefore new alternatives need to be used, such as immunotherapy targeting specific receptors of malignant cells. Among all the options, CAR (Chimeric antigen receptor)-based therapy has arisen as a new opportunity for refractory or relapsed hematological cancer patients. CARs were designed to be used along with T lymphocytes, creating CAR-T cells, but they are presenting such encouraging results that they are already in use as drugs. Nonetheless, their side-effects and the fact that it is not possible to infuse an allogenic CAR-T product without causing graft-versus-host-disease, have meant using a different cell source to solve these problems, such as Natural Killer (NK) cells. Although CAR-based treatment is a high-speed race led by CAR-T cells, CAR-NK cells are slowly (but surely) consolidating their position; their demonstrated efficacy and the lack of undesirable side-effects is opening a new door for CAR-based treatments. CAR-NKs are now in the field to stay.

## 1. Background

B cell leukemias, such as Acute lymphoblastic leukemia (ALL) and Chronic lymphocytic leukemia (CLL), are hematological cancers affecting young and elderly patients [1]. In a patient with ALL, large amounts of stem cells become B-lymphoblasts very quickly, so the function of leukemia cells is then compromised and they are unable to fight infection correctly. Furthermore, leukemia cell numbers increase in both blood and bone marrow to such a high level (100 or even 1000 times more than normal); as a consequence healthy white blood cells, red blood cells and platelets are reduced, leading to anemia, infection and easy bleeding [2]. The current protocol in the diagnosis and classification of ALL involves the study of its morphology, immunophenotype, cytochemistry, cytogenetics, and molecular genetics [3]. Particularly, the immunophenotype is vital for the initial diagnostic work-up of ALL. In all B-lineage ALLs, total expression of the CD19 surface marker is mainly present, along with the total or partial expression of other surface markers such as CD22, CD20 and CD33. The median content of blast cells in bone marrow is around 82% [4]. ALL is the most prevalent cancer in children, with a rate of 20–25% of all cases [5], but this disease has become highly curable, with five-year survival rates above 90% [6]. In contrast, the outcome in adults is quite different, with a five-year survival rate of 40% with conventional treatments. Prognosis is more adverse with age, and this may be due to more unfavorable cytogenetic/genetic anomalies and patient comorbidities [7].

In adults, CLL is one of the most frequent types of leukemia, which usually evolves moderately slowly. It is a low-grade lymphoproliferative neoplasm with ≥5 × 10^9^/L clonal B-cells in the peripheral blood, which usually express CD19, CD20, dim CD23, and CD5 [8]. It generally manifests during or after middle age and is exceptional in children. In CLL, a large number of hematopoietic stem cells turn into anomalous lymphocytes and do not differentiate into healthy white blood cells; as a consequence, the lymphocytes are not able to fight infection appropriately. Moreover, CLL patients do not normally show any symptoms and are often diagnosed during a routine blood test [9]. The five-year survival rate for CLL patients is 79.2%, and it is still incurable in many patients [10]. Furthermore, a major complication of CLL is the transformation into a fast-growing type of non-Hodgkin Lymphoma (NHL) called diffuse large B-cell lymphoma (DLBCL). This is known as Richter’s transformation, and it happens in 2% to 10% of CLL cases. Rarely, in CLL patients, the leukemia can also transform into ALL, and when this occurs, the treatment administered to ALL patients is likely to be used in CLL patients as well [11].

## 2. Standard Therapies and Treatment Improvements against Hematological Cancers

### 2.1. Standard Therapies for ALL

Over the last four decades, ALL treatment advances have principally been made in the children and adolescent populations, with less success reported in adults [12,13] ALL patients are mainly treated with chemotherapy, and treatment is usually split into three phases: induction, consolidation and maintenance. The aim of induction chemotherapy is to bring the leukemia to the point where it lacks detectable tumor cells (complete remission). There are several chemotherapy drug combinations for the induction phase, which generally include dexamethasone, vincristine or prednisone, and an anthracycline drug such as daunorubicin or doxorubicin (Adriamycin), and L-aspaginase. Next, the aim of the consolidation phase is to eliminate any leukemia cells that may still be in the blood or bone marrow but are undetectable in tests. This reduces the risk of the leukemia relapse, and it often includes another short course of high doses chemotherapy, using methotrexate (MTX) and cytarabine (ARA-C). Lastly, after consolidation, in most cases the patient receives a chemotherapy maintenance regime of methotrexate and 6-mercaptopurine (6-MP) with the aim of ALL in remission. Occasionally, this may be given along with other drugs such as prednisone and vincristine. However, 10–20% of ALL patients are refractory, which means that the leukemia cells do not disappear with the first treatment. Next, different or higher doses of chemotherapy drugs may be used, such as clofarabin, even though the outcome is poorer [14,15,16,17]. Other drugs such as blinatumomab (Blincyto) [18], rituximab or inotuzumab ozogamicin (Besponsa) may be a choice for ALL patients [19]. A hematopoietic stem cell transplant (HSCT) may be tried when the ALL is put into remission. Minimal residual disease is a key variable for the HSCT outcome [20], since if the ALL enters into remission with the first treatment but then relapses, bone marrow and blood will be affected. The chosen treatment depends on the time elapsed from the initial treatment until the leukemia returns. In the event of a long time free of disease, a similar treatment may be tried for a second remission. However, if the relapse appears earlier, new drugs for more combative chemotherapy may be used [21,22].

There are two principal types of HSCT: allogenic stem cell transplants, in which the stem cells belong to a donor (bone marrow, blood or umbilical cord blood (CB), which is the preferred transplant for ALL patients; and autologous stem cell transplants, in which the patient receives their own blood cells.

### 2.2. Standard Therapies for CLL

A greater number of CLL patients have no symptoms at diagnosis, and all patients should be classified based on their risk. Patients classified as low-and intermediate-risk (~75% patients) should be checked for disease progression every half a year to a year, while those in the high and very high-risk group (~25% patients) should be checked every 3–6 months [23]. For the initiation of a treatment against CLL, patients must present any of the following symptoms: (a) progressive marrow failure, (b) progressive lymphocytosis, (c) massive nodes with or without progressive or symptomatic lymphadenopathy or (d) autoimmune complications of CLL [24]. Patients could also present clinical symptoms such as fever, asthenia or night sweats. Treatment choices for CLL differ widely, determined by the patient’s age, their risk group classification, and the different symptoms that led to treatment. Many patients live with CLL for a long time, but usually this disease is extremely hard to cure, and it has been proved that early treatment helps prolong life. Patients without significant comorbidity are treated with rituximab, fluradabine and cyclophosphamide; patients with comorbidity are treated with obinutuzumab-chlorambucil or rituximab-chlorambucil. Finally, patients who present deletions in 17p or alterations of p53 are treated with ibrutinib, idelalisib, ofatumumab or venetoclax [25].

**Figure 1:**
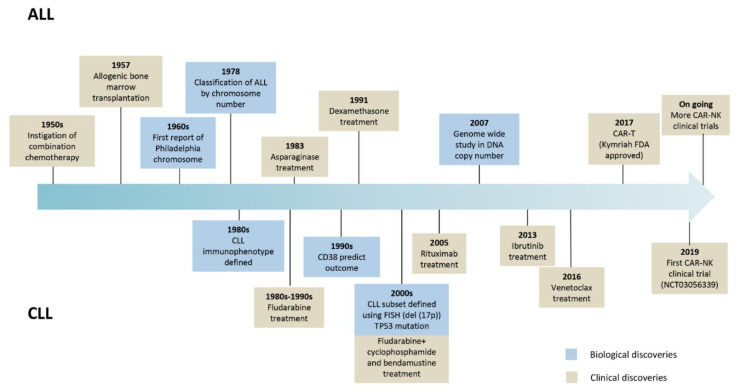
Landmark advances in the evolution of biological and clinical research in ALL and CLL.

Relapsed or refractory ALL or CLL patients after two or more lines of treatment may need alternative therapies to treat their disease. New therapies, especially immunotherapies, are emerging to treat these and other hematological cancers. Chimeric antigen receptor (CAR) based therapy is positioning itself as one of the most promising therapies for getting rid of the malignant cells. ALL and CLL biological and clinical milestones are summarized in Figure 1.

### 2.3. Immunotherapies against ALL and CLL

Immunotherapy is a type of therapy that uses substances to stimulate or suppress the immune system to help the body fight cancer, infection, and other diseases [26,27]. The 2018 Nobel Prize in Physiology or Medicine was awarded to Prof. James P. Allison and Prof. Tasuku Honjo for their achievements in cancer immunotherapy by inhibition of negative immune regulation. Several types of immunotherapies are used to treat cancer, and these treatments can either help the immune system attack the cancer directly or stimulate the immune system in a more general way. Indeed, prior to its designation as the Science Breakthrough of the Year in 2013, cancer immunotherapy was already active in the treatment of hematologic malignancies [28].

In ALL, complete remission (CR) rates after chemotherapy are low and range between 25 and 45% [29], with most of these patients dying, which leaves a lot of room for improvement. Generally, four different immunotherapies have been established to date, including conjugated monoclonal antibodies, naked monoclonal antibodies (mAbs), bispecific T cell engager (BiTE), and chimeric antigen receptor (CAR) T cell therapy [30]. These therapies can target different antigens present on the surface of B cells. 

About 30 to 50% of late pre-B cell lymphoblasts express CD20 and it is linked to a higher relapse and lower survival rate [31,32]. Several drugs that target this receptor are used in the clinic, such as rituximab, which has been integrated into chemotherapy programs [33]; ofatumumab, which induces higher levels of Complement-dependent-Citotoxicity (CDC) and Antibody-Dependent-Cellular- Cytotoxicity (ADCC) compared to rituximab [34]; and obinutuzumab, which is designed to intensify ADCC as compared to ofatumumab and rituximab [35]. Although the Food and Drug Administration (FDA) approved it for first-line treatment of CLL, the clinical scene of pre-B ALL needs to be studied. 

CD22 is present on leukemic blasts in >90% of ALL patients [36]. Mainly, two drugs are used in ALL for targeting the CD22 antigen, both monoclonal antibodies: epratuzumab, as an orphan drug [37] and inotuzumab ozogamicin (InO) [38].

CD19 is expressed in 90% of pre-B and mature ALL lymphoblasts, providing an interesting target for immunotherapy. A BiTE construct named blinatumomab binds CD19-positive B cells and CD3-positive cytotoxic T cells. The cytotoxic T cells are activated when binding to CD19 and induce cell death via direct tumor lysis [39]. In October 2017 the FDA approved one of the most promising cellular therapy-based treatments for relapsed B-cell ALL: Tisagenlecleucel (Kymriah from Novartis). A short time later, Axicabtagene Ciloleucel (Yescarta from Kite-Gilead) was approved for relapsed or refractory large B cell lymphomas. In 2020, a third treatment named Brexucabtagene Autoleucel (TECARTUS, Kite-Pharma) was approved by the FDA. These treatments are Chimeric antigen receptor (CAR) T-cell based therapies [40], the first two of which were approved in Europe by the European Medicines Agency (EMA) in June 2018, and the third, TECARTUS, was approved in December 2020. 

With CLL, in most cases several immunotherapies are applied to the patients as a first-line therapy. These antibodies, such as Rituximab, Ofatumumab or Obinutuzumab, target the CD20 pathway [23], while a monoclonal antibody that binds to CD52 (alemtuzumab) targets CD52 present in lymphocytes for destruction. This immunotherapy is usually administered to patients previously treated with alkylating agents, and after failure of fludarabine therapy [41]. All these therapies are summarized in Table 1.

## 3. State of the Art of CD19-CAR-T Therapies. From Bench to Current Clinical Trials Results

In the 1980s, Israeli researchers expressed chimeric TCR genes including the TCR constant domains united to the variable domain from an antibody, which led to a hypothesis about CAR-T treatments [42]; in 1989, Gideon Gross and Zelig Eshhar developed the first CAR-T cells at the Weizmann Institute, Israel. Some years later, Prof. Carl H. June from the University of Pennsylvania tested genetically modified CAR-Ts in humans for the treatment of cancer and clinical use, and thanks to his work, the first FDA-approved gene therapy, named Tisagenlecleucel (Novartis), was developed and commercialized. A CAR is a chimeric receptor construct consisting of an extracellular single-chain variable fragment (scFv) derived from an antibody [43] or a full-length antibody [44]. It is connected to a hinge fragment, which acts as a “spacer” between the extracellular and intracellular part, being usually a CD8α, which enhances responses initiated by TCR [45]; a transmembrane domain, and a CD3 ζ chain, or FcR receptor γ, consisting of an intracellular tyrosine-based activation motif. This was the structure of the first generation CARs (1G) [46]. T cell activation could be mediated by TCR ligation of the host antigen. Two signals are needed to activate the T cells fully. One of the signals is through the TCR, whereas the second signal is activated by the recognition of CD86 or CD80 in the surface of antigen presenting cells (APC), co-stimulating CD28. Consequently, during infection or inflammation, APCs upregulate CD86 and CD80 and both signals, TCR and CD28, are activated, so that T-cells perform target killing, with long-term persistence [47,48]. Researchers accordingly designed a CAR which included the two-signal model of T-cell activation CD28 co-stimulatory domain along with CD3ζ ITAM domains [49]. These kinds of constructs constitute second generation CARs (2G). Furthermore, it has also been reported that other co-stimulatory domains, such as 4-1BB, support comparable in vivo improvements to CAR-T cell persistence and function [50]. Nonetheless, CAR-T cell properties could change in regard to these last two domains; CD28-based CARs have direct antitumor efficacy, while 4-1BB-based CARs have long persistence activity [51]. As a result, third generation (3G) CARs have been developed to include two co-stimulatory domains, 4-1BB and CD28 intracellular domains [52]. CARs from 2G or 3G containing the 4-1BB domain have been reported to have greater in vivo expansion and anti-tumor activity compared to CD28 2G CARs [53]. Due to the vast heterogeneity of cancer cells in solid tumors, a fourth generation of CARs (4G), known as TRUCKs (“T cells redirected for antigen-unrestricted cytokine-initiated killing”) were developed, where cytokines are used to armor CARs. These CARs contain an additional modification which consists of an inducible or constitutive expression cassette for a transgenic protein, for example a cytokine, which is released by the CAR-T cell to modulate the T-cell response; As a consequence, an improvement of T cell properties and recruitment of additional immune cells can be achieved [54].

As CARs seem to be a newer and more effective way to treat cancers in relapse or refractoriness, especially hematological cancers, there are various clinical trials going on. Most of them use T cells as a vehicle for the CARs, with more than 1.000 clinical trials worldwide, and CD19 is mostly used as the CAR antigen.

## 4. The Role of NK Cells in the Immune System

Natural Killer cells, or NK cells, belong to the innate immune system, providing rapid responses against viral infections and tumors. Usually, the detection of the major histocompatibility complex (MHC) on the surface of the infected cells by the immune cells triggers cytokine release, causing lysis or apoptosis. NK cells have, in fact, a unique ability to recognize stressed cells lacking antibodies and MHC, accelerating an immune reaction. They owe their name “natural killers” to the initial perception that they do not need prior activation to kill cells with no “self” antigens of MHC class I. As malignant cells do not express MHC I markers, T cells cannot destroy them, so NK cells play a key role [55].

NK cells and their functions were described more than 30 years ago, but for the first time in 1975 these cells were described as bigger lymphocytes than B cells and T cells, which contained distinctive cytoplasmic granules. NK cells were characterized as cells which showed a co-stimulatory independent spontaneous cytotoxic capacity, differentiating them functionally from B cells and T cells [56,57].

In the immune system, NK cells are the third major lymphocyte subset. These large granular cells constitute approximately 10–15% of lymphocytes in the blood [58]. NK cells are able to kill tumor cells and infected cells “naturally”, i.e., in a casual manner that does not need any prior activation and is not limited to the expression of MHC molecules [57,59]. NK cells are usually defined within the lymphocyte population by a lack of CD3 and expression of CD56, a neural cell adhesion molecule (NCAM) [60].

During the development process of NK cells, they express several surface markers progressively and in an orderly way, and they are classified into stage 1 (CD34+, CD45RA+, CD117−, CD94−, CD56−, CD16−), stage 2 (CD34+, CD45RA+, CD117+, CD94−, CD56−, CD16−), and stage 3 (CD34− CD117+, CD94−, CD56−, CD16−). Mature NK cells are phenotypically described as stage 4 (CD34−, CD94+, CD117+/−, CD56bright, CD16+/−) and stage 5 (CD34−, CD94+/−, CD117−, CD56dim, CD16+) [61].

NK cell functions involve recognition of potential target cells by the initial binding interactions between activating and inhibitory receptors with ligands available on the target, and the integration of signals transmitted by these receptors, which determines whether the NK cell detaches and moves on or stays and responds. Therefore, the activating and inhibitory receptors are crucial for NK cell function regulation. NK cells express clonally distributed inhibitor receptors named killer cell immunoglobulin-like receptors (KIRs), that recognize allotypic determinants (KIR ligands) shared by particular groups of HLA class I alleles. The regulatory mechanism mediated by these receptors is thought to protect normal cells from autologous NK cell attack, while rendering cells for which class I expression is compromised (e.g., by tumor transformation or viral infection) susceptible to NK-mediated killing [62]. The absence of HLA molecules in the membrane is not enough to trigger a response in NK cells. A larger number of activating signal are needed. Several different families of activating receptors are found in NK cells. C-type lectine family, mainly represented by NKG2C and NKG2D receptors, which interact with DAP10 and DAP12 [63,64]. Other activating receptors are NCR family, practically exclusive in NK cells, mainly form by NKp30 (CD337), NKp44 (CD336) and NKp46 (CD335) [65]; and SLAM family, in which we found 2B4 (CD244) [66]. The imbalance between inhibitory and activating signals will determine the killing outcome of NK cells.

NK cell role during ALL or CLL may determine the prognosis of the disease. An increase in the number of NK cells is associated with a better prognosis in ALL and CLL [67]. The presence of NK cells in bone marrow is associated with better prognosis and higher chances of a good response to chemotherapy in ALL patients [68]. Besides, a strong NK cell phenotype at the time of ALL diagnosis seems to be related with the control of the disease after chemotherapy treatment [69]. In CLL, the role of NK cells remains controversial. While it was described that CLL patients paired with defects in NK cell cytotoxicity [70], other groups demonstrated that NK cell functions are not affected in CLL patients [71]. Thus, the role of NK cells is still uncertain.

## 5. Advantages and Disadvantages of CAR-NK and CAR-T Therapies

**Figure 2:**
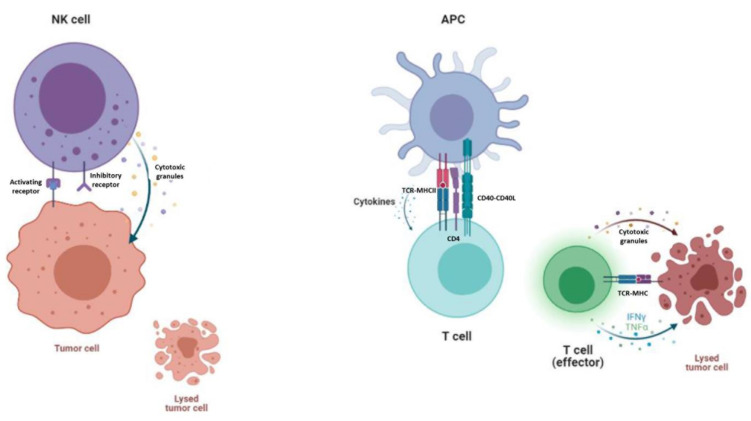
Mechanism of action of NK cells and T cells. On the left, NK cells are killing a tumor cell, as inhibitory receptors do not encounter self-recognition in other cells. NK cells secrete granulocytes such as perforin and granzyme in order to kill the tumor cell. On the right, a T cell is being primed for activation by APC and cytokine stimulation. An effector T cell kills the tumor cell by secreting granulocytes and other cytokines into the tumor microenvironment.

Despite the good results of CAR-T cells, there are some expected side-effects. On the one hand, the main and most serious side-effect of CAR-T cell therapy is cytokine-release syndrome and its dangerous form, a “cytokine storm”, in which T-cells are massively activated, triggering a cascade of pro-inflammatory cytokines which cause flushing, fever and dyspnea. Although an acute cytokine storm can potentially be lethal, it has been proved that the anti-interleukin-6 receptor antibody tocilizumab is an effective treatment [72]. Immune effector cell associated neurotoxicity syndrome (ICANS) has also emerged as a serious side-effect after CAR-T cell therapy. On the other hand, as these patients are usually highly medicated, it is not always viable to expand and manufacture their own autologous modified T-cells from lymphocytes, due to the scarce lymphocyte count or the poor state of the cells. Hence, the manufacture of off-the-shelf allogenic CAR-T cells from healthy donors’ lymphocytes is promising in many aspects, although there are some concerns that keep them from use in clinical trials [73]. Allogenic T cells express the human leucocyte antigen (HLA), which can give a mismatch between donor and recipient, leading to severe, even lethal graft-versus-host disease (GvHD) [74]. This leads to a new source of cells, since the less alloreactive T cell subset such as CD45RA^-^ lymphocytes [75] and Natural Killer (NK) cells are good candidates because they suppress GvHD by inhibiting activated, alloreactive T cells without causing GvHD themselves [76,77]. NK lymphocytes constitute an attractive source for CAR-based treatments, owing to their innate ability to kill malignant or infected cells without prior activation or HLA restriction [78]. Moreover, due to NK cells’ shorter lifespan, B cell depletion could be less severe for the patient [79]. However, T cell expansion occurs in a differential manner regarding their subpopulations and polyclonal populations [80]; this could lead to poor expansion and persistence, which is directly correlated with patient relapse [81]. Another point to address is that, with a good initial cell product, T cells are easier to expand and less resistant to genetic engineering, thanks to the use of a CD3/CD28 activation kit [82]. Although NK cells appear to be harder to expand and transfect, some groups have achieved impressive fold expansion numbers by co-culturing them with activation beads or modified feeder cells [83,84] along with some good transduction numbers [85]. The mechanism of action of NK cells differs from that of T cells. On one hand, NK cells interact with target cells through activating and inhibitory receptors, and the outcome is determined by the accumulation of signal strength. If they are activated, they release cytotoxic granules, such as perforin and granzyme, and they secrete a variety of cytokines [86]. T cells, however, are activated through antigen presenting cells (APC). This triggers a signalling cascade from the TCR complex, which transforms the T cell from a resting state to a state of activation and proliferation [87]. T cells also need co-stimulation from APC and cytokines in order to attack tumor cells [88] (Figure 2).

Finally, T cells and NK cells are differentially activated. Despite the fact that some of the signal domains are conserved between these two types of lymphocytes, such as CD3ζ and 4-1BB, other co-stimulatory domains typically present in T cells are absent in an NK cell, such as CD8α and CD28 [89]. NK cells can operate through several adapter domains for downstream signaling, such as CD3ζ, DAP10, DAP12, and FcRγ chains. While CD3ζ signaling occurs via CD16, NKp30, and NKp46, FcRγ chains signalling also occurs via CD16, NKp30 and NKp46. DAP10 mediates signaling through NKG2D whereas DAP12 activates KIRs, NKG2C, and NKp44 [90,91]. These differences between CAR-T and CAR-NK therapy are shown in Table 2.

## 6. Designing NK Cells Specific CAR Constructs

**Figure 3:**
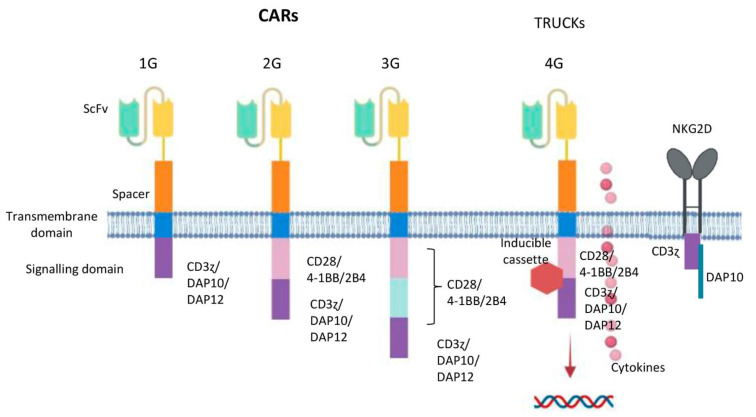
Potential design of CAR generation according to NK cell intracellular motifs and functions. From left to right: First generation CARs (1G) have an intracellular tyrosine-based activation motif, such as a CD3 ζ chain, DAP10 or DAP12. Second generation CARs (2G) include a co-stimulatory domain (CD28, 4-1BB or 2B4) in tandem with CD3ζ or DAP10 or DAP12. Third generation CARs (3G) contain two co-stimulatory elements (CD28, 4-1BB or 2B4) in tandem with CD3ζ or DAP10 or DAP12. Fourth generation CARs (4G or TRUCKs) are additionally modified with an inducible or constitutive expression cassette for a transgenic protein. NKG2D ectodomain with DAP10 and CD3ζ cytoplasmic signalling endodomain.

As mentioned before, NK cells can be activated through CD3ζ, resulting in ADCC mediated by CD16 receptor. Thus, the vast majority of CAR constructs used for engineering NK cells contains this signaling component [92], classically present since 1G CARs. Despite the fact that traditional 2G CARs designed for T cells, that is, CARs containing CD3ζ and CD28 or 4-1BB domains, are functional in NK cells [85] new approaches need to be explored for NK cells (Figure 3). Taking into account other main signaling pathways that activate NK cells, new CAR constructs have been designed, in which signaling domains derived from 2B4, NKG2D, DAP10 or DAP12 have shown some promising results [93,94,95,96]. A 2B4 containing CAR construct integrated in the endodomain significantly enhanced all aspects of the NK-cell activation, having a powerful costimulatory effect in NK cells [96]. Regarding NKG2D, coupling this ectodomain with DAP10 and CD3ζ cytoplasmic signaling endodomain has shown increased NKG2D expression on CAR-NK cells with enhanced cytotoxicity against tumor cell lines [97]. Nevertheless, including the DAP10 cytoplasmic endodomain seemed to be needless since CAR-NK cells without DAP10 outperformed those with the DAP10 domain [98]. When designing a new CAR, the active interaction between the transmembrane domain and the endodomain must be taken into consideration to guarantee functionality of the CAR construct. Accordingly, the design of a perfect CAR for NK cells is still a current challenge in which a deep understanding of NK cell and CAR design signaling is crucial to enhance potency and performance in vivo [99].

## 7. NK Cells from Several Cell Sources: Adult Peripheral Blood, Umbilical Cord Blood, Hematopoietic Progenitors from Cord Blood and Human-Induced Pluripotent Stem Cells

**Figure 4:**
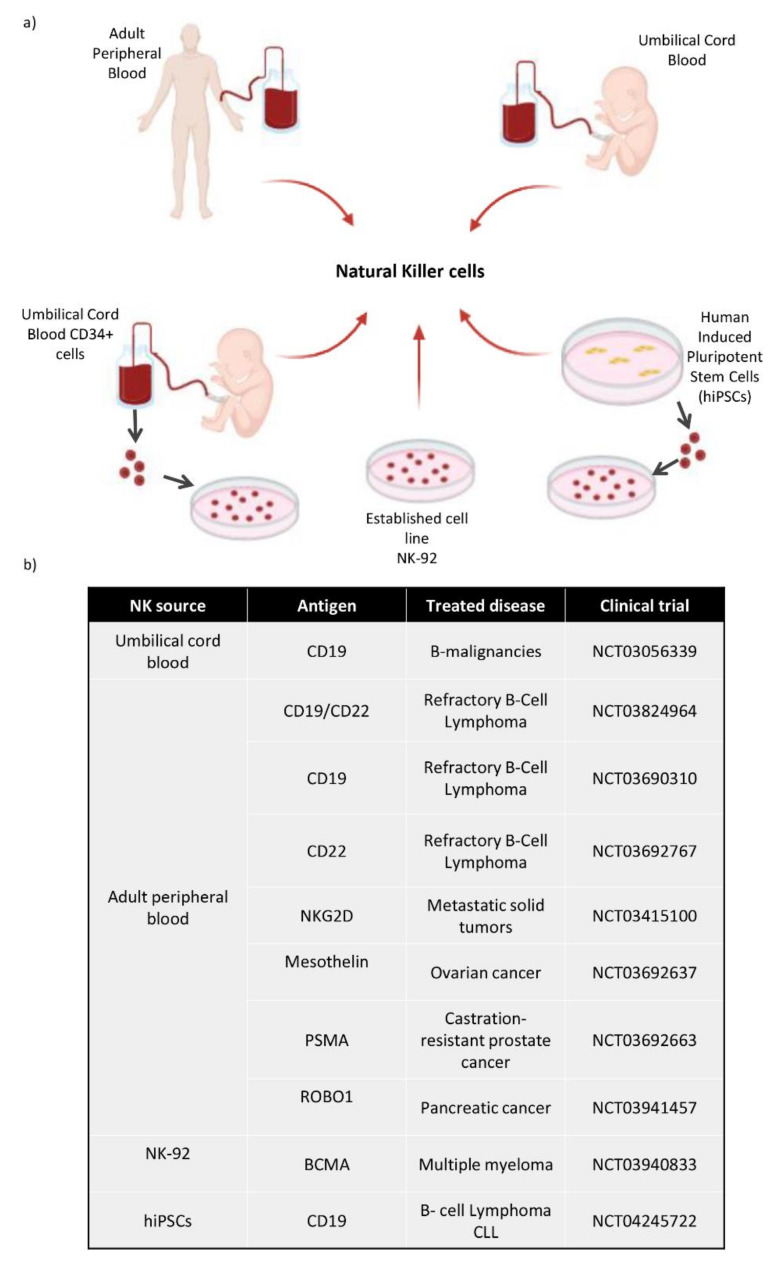
(**a**) Diagram of the different cell sources used to obtain NK cells. These NK cells can be directly collected from Adult Peripheral Blood and Umbilical Cord Blood. They can be differentiated in vitro from CD34+ hematopoietic stem cells taken from umbilical cord blood, and also from hiPSCs, which are differentiated towards CD34+ cells and then to NK cells. There is an established NK cell line called NK-92. (**b**) Most relevant CAR-NK clinical trials use different sources of NK cells and different antigens, depending on the type of cancer.

NK cells can be obtained from different cell sources as shown in Figure 4a. Firstly, NK cells can be isolated from adult blood (AB) or cord blood (CB) PBMCs by negative selection after magnetic cell isolation. NK cells can be cultured with different cytokines, such as IL-2 and/or IL-15 in order to ensure their survival, proliferation and higher cytotoxicity [100]. We can obtain fully mature and functional NK cells from these cell sources, although their number is limited and hard to expand. Secondly, among all the uses that could be attributed to human induced pluripotent stem cells (hiPSCs), the generation of hematopoietic stem cells is one of the most widely studied, and several protocols have been proposed for getting in vitro CD34+ cells [101,102,103]. Firstly, it requires CD34+ hematopoietic precursors to be obtained for differentiating protocols from human embryonic stem cells (hESCs) and hiPSCs. There are several stromal cell lines used in co-culture systems with hESCs/hiPSCs; for instance, OP9 cells are the most popular [104]. In the first data reported, they were able to obtain up to 20% of CD34+ cells by coculturing hESCs with OP9 cells.

Therefore, these in vitro generated hematopoietic stem cells could be used to obtain different cells from the hematopoietic lineage, such as T cells [105], platelets [106], red blood cells [107] and NK cells. HiPSCs could became a new source for immunotherapies involving NK cells, as several groups have developed methods for producing clinical scale NK cells from hiPSCs [108,109]. Moreover, as previously mentioned in the Natural Killer cells clinical trials section, a hiPSCs derived NK cell pharmacology product was developed for treating several solid tumors (FT500) in 2019. Not only did they create this product, but they also used the hiPSCs derived NK cells for CAR based treatments (FT596).

Finally, hiPSCss constitute a source for NK cells, as do CD34+ cells from umbilical cord blood, since these cells could have therapeutic uses apart from hematopoietic stem cell transplantation [110,111]. They have been taken into consideration, as they could provide a large number of NK cells [112,113], already extensively described [114]. In general, all NK cell sources mentioned above can provide fully mature and functional NK cells suitable for immunotherapy, but the NK cells with high rates of proliferation described are NK cells from CD34+ cells and hiPSCs cells sources in comparison with peripheral and umbilical blood sources.

## 8. State of the Art of NK Cell Therapies and CD19-CAR-NK Therapies with the Recent Clinical Trial Data in Refractory B Malignancies Patients

There are several clinical trials taking place to treat different types of cancer with NK cells; combinations of cryosurgery and NK-based immunotherapy for advanced kidney cancer (NCT02843607), NK cell-based immunotherapy as maintenance therapy for small-cell lung cancer (NCT03410368) or NK cells along with IL-2 following chemotherapy to treat advanced melanoma or kidney cancer (NCT00328861). hiPSC-derived NK cells, named FT500, are also being used in combination with Immune Checkpoint Inhibitors (ICI) in a clinical trial to treat subjects with advanced solid tumors (NCT03841110). In April 2019, Fate Therapeutics declared that the first patient treated with FT500 had successfully completed an initial safety appraisal. The patient was administered with three once-weekly doses of FT500. There were no toxicities or severe adverse events reported and the treatment cycle was well tolerated, with no dose-limiting toxicities or severe adverse events reported during the initial 28-day observation period. Not only are NK cells able to treat solid tumors, but they also play a key role in immunotherapies against hematological cancer like acute myeloid leukemia (AML), by using high doses of these cells [115] or infusing NK cells after chemotherapy along with IL-2 (NCT02763475). Nonetheless, expanded and stimulated NK cells or high-dose NK cell therapy are not the only options when treating patients.

Although T cells have typically been used in CAR technology-based therapy, with more than 400 clinical trials on-going and 3 commercial products, Kymriah Yescarta and TECARTUS, NK cells are also emerging as one of the new promises in this field [116], as shown in Figure 4b. Due to their low infection rate, poor in vivo expansion and short life span, NK cells were not taken into account from the beginning for this kind of therapy. Nevertheless, newer protocols that enhance viral transduction efficiency and prosperous expansion of these cells have made a space for NK cells in the CAR therapy field [84]. Furthermore, allogenic NK cells have a major advantage over allogenic T cells, i.e., they could be used as a “universal” product as they do not cause GvHD as they lack TCR [76]. The interest in using allogenic NK cells for this kind of therapy is increasing, and there are already 13 clinical trials using CD19 CAR-NK cells (NCT03056339, NCT03690310, NCT00995137, NCT01974479, NCT04639739, NCT02892695, NCT04887012, NCT04796675, NCT03824964, NCT05020678, NCT03579927, NCT04796688, NCT02134262), and numerous preclinical studies with NK cells from different sources as vehicles. Firstly, NK-92 is an established NK cell line of a non-Hodgkin’s lymphoma patient [117]. NK-92 cells, featured with activated human NK cells, are applied in clinical practice for allogenic adoptive cellular immunotherapy. Due to the loss of absent expression of most currently known KIRs, NK-92 cells target and kill a wider range of tumor cells with enhanced toxicity in vitro and in vivo [118]. The NK-92 cells are irradiated before infusion, avoiding the induction of NK-derived leukemia. Despite the fact that the NK-92 cell line has been proved safe for clinical use [119], however, these cells are not as consistent as expected at killing lymphoid blast by themselves [120]. Taking this into account, the use of a CAR along with these cells could successfully kill NK-resistant lymphoblastic leukemia cells. Several preclinical studies confirm that NK-92 cell lines are a good source for CAR based therapy, as they possess consistent cytotoxic activity, a good expansion rate and low tumorigenicity risk when irradiated and transfused in patients [121,122,123].

Secondly, allogenic primary NK cells from adult peripheral blood (AB) or umbilical cord blood (CB) could represent feasible, safe, off-the-shelf CAR-cell products to treat various malignancies such as hematological cancers. When studying AB CD19-CAR-NK cells, they not only successfully kill CD19-expressing target cells, but they also retain the function and expression of their native activating receptors, preserving their activity [83]. However, AB NK cells are more variable from donor to donor in number, and they expand and activate less in vitro than CB NK cells [85,124]. Some studies show higher antitumor activity of CB cells compared with other NK cell sources, which justifies the use of CB-derived immunotherapy [125]. Moreover, CB units stored in blood banks could be used for this purpose. CB CAR-NK cells have shown great performance against their target cells, and more flexibility to be expanded [84]. These CB CAR- NK cells are currently being used in a clinical trial at MD Anderson Cancer Center targeting CD19 cells, with great results. CAR-NK cells from cord blood were administered to 11 patients with relapsed or refractory CD19-positive cancers (NHL or CLL). An anti-CD19-CD28-CD3ζ CAR was used for the transduction and the retroviral vector included an IL-15 gene and a suicidal switch. Seven out of eleven patients achieved a complete remission, exhibiting a significantly higher early expansion of CAR-NK cells compared to the non-responders. There were no reported side-effects associated with the high response rate of the treatment, even in even in KIR-ligand mismatch cases (5/11), and there was no interleukin-6 increase, which proved the safety of the treatment. The published observation of circulating CAR-NK cells by flow cytometry was limited to the first three weeks [126].

Finally, in the last few years, hiPSCs have arisen as one of the most promising cell sources for personalized medicine. HiPSC-NK cells can be manufactured from standardized cells resulting in a homogeneous clinical scale NK cell population [78]. These standardized NK cells would be excellent candidates for CAR based treatment as they show a similar phenotype and similar anti-tumor activity to AB NK cells [127]. As a matter of fact, iPSC-derived, universal, off-the-shelf CAR-NK cell immunotherapy for B-cell hematological cancer has been manufactured as a product called FT596. This product is being used in a phase I clinical trial (NCT04245722) along with anti-CD20 monoclonal antibodies. FT596 product was administrated to 20 patients in order to evaluate for assessment of safety and efficacy in the first, second and third dose cohorts. From the 14 patients that were administrated with a second and third dose, 10 of them achieved an objective response [128].

Nowadays the clinical indications of CAR-NK cell therapy in B cell hematological cancer remains an open question. Currently, it has been confirmed that various CAR-T cell therapy clinical trials are safe being an efficient therapeutic procedure for relapse or refractory B-ALL patients when used as a bridging approach before or after HSCT. Therefore, CAR-NK cells may also handle as a bridge strategy, through which patients can accomplish a low pre-infusion minimal residual disease (MRD) condition before administration of allo-HSCT. The aim of NCT02892695 clinical trial is to figure out the safety and best dose of CD19 CAR-NK cells used as a bridge therapy in patients who intend to undergo HSCT [129].

Other potential uses of CAR-NK cellular therapy may be indicated for patients that have not enough T cell numbers for autologous CAR-T cell product manufacture. Patients with aggressive ALL or CLL will benefit from allogeneic CAR-NK permitting early treatment or later in combination with CAR-T. Additionally, for those CD19+ patients that have been relapsed after CAR-T therapy or patients who develop high toxicity (ICANS and/or cytokine-release syndrome) after CAR-T infusion.

## 9. Challenges and Future Perspectives

Due to the boom in immunotherapy for hematological cancers, great strides have been made in this field, especially with the arrival of CAR-T treatments. However, despite the successful results obtained with autologous CAR-T treatment, there are still some worrying aspects. Undesirable side-effects, such as cytokine storms, ICANS or GvHD caused by allogenic CAR-T cells, are driving the CAR field towards new alternatives. The lack of side-effects or GvHD in allogenic treatments has put NK cells in the spotlight.

For the last few years, CAR-NK cells have proved to be an optimum product for the treatment of hematological malignances *in vitro.* Recently, the report from one of the few clinical trials taking place has not only proved their efficacy but also their safety when using allogenic CAR-NK cells. Hence, CAR-NK cells can serve as an off-the-shelf product to treat refractory hematological malignances, with no need for the patient-specific product request by CAR-T treatments. Moreover, the possibility of obtaining in vitro generated NK cells for this purpose could lead to massive production of universal allogenic CAR-NK cells, which could end up in a reduction of costs and more open access to this treatment. Combinations of CAR-NK products with other immunotherapies and even with other CAR-T cells are becoming a promising option.

Therefore, the benefits of CAR-NK cells over CAR-T cells augur promising applications in cellular immunotherapy against hematological malignancies as an alternative or combination cell drug. Nevertheless, there are still challenges to be addressed. For instance, because of the heterogeneity of NK cells with various functional features, the selection of appropriate NK cell subsets (e.g., killer, naïve or memory cell subsets) to specifically expand and arm CAR-NK cells has to be explored. NK cells are known for their transduction difficulty. Although several protocols with retrovirus and lentivirus have been successfully developed, there is still room for new techniques in order to improve NK cell transduction, such as mRNA electroporation or CRISPR/Cas9 technology. As CAR-NK cells are thought to be an “off-the-shelf” product, cryopreservation is also a crucial step. Focusing on not losing many NK cells at the thawing process, and re-establishing their function, it is determinant to elucidate the appropriate cryopreserving media and protocol for CAR-NK cell therapy success. Due to their short persistence in vivo, the NK cell cytolytic effect could also be restricted, but they are probably unable to trigger cytokine storms or on-target/off-tumor effects; continuous cytokine support or several infusions of CAR-NK cells may will be needed. Additionally, various NK cell sources have been studied (peripheral blood, cord blood or hiPSCs), and therefore in the future the foremost appropriate one could be used for refractory malignancies. Moreover, the best configuration of CARs to boost the activation, proliferation, cytolytic activity and cytokine secretion of NK cells has not yet been found. Perhaps the future of these CAR constructs for NK cells lies on exploring NK cell activating domains such as NKG2D, DAP10, DAP12 and 2B4 in order to improve their performance. Moreover, future directions of CAR-NK cell therapy could be administrated in combination with other therapies, being lymphodepletion, radiation, immune checkpoint blockers or even CAR-T cells. Notwithstanding, as a result of the recent advances, quick developments and future challenges for improvement, CAR-NK cell-based immunotherapy constitutes an encouraging scenario for cancer treatment. Tumor-associated antigens do not require the recognition of HLA molecules from patients, which makes it possible to manufacture off the shelf NK cell banks rather than manufacturing individualized CAR-NK cells. In conclusion, as more evidence from clinical trials is procured within the coming years, CAR-NK cell therapies could provide meaningful progress in tumor immunotherapy. Moreover, CAR-NK therapies in combination with other immunotherapies or even other CAR-T cells may pave a new way for CAR-NK cell-based immunotherapy in the future.

## 10. Conclusions

CAR-NK cells have excellent potential as an innovative and “off-the-shelf” cellular immunotherapy against hematological cancer that could be quick, accessible and safe for clinical practice. With the growing safety and encouraging work reported in preclinical studies and clinical trials, together with progressive achievements addressing the remaining challenges, it is envisioned that CAR-NK cell therapy will progress to emerge and contribute to significant improvement in the survival of relapsed or refractory hematological cancer patients.

## Figures and Tables

**Table 1:** Different immunotherapies used for treating ALL and/or CLL.

Target	Drug	Immunotherapy Type	Use and Clinical Indication
CD19	Blinatumomab [39]	BiTE	ALL
	Tisagenlecleucel [40]	CAR	ALL
	Brexucabtagene Autoleucel [40]	CAR	ALL
CD20	Rituximab [33]	Monoclonal Ab	ALL, CLL
	Ofatumumab [34]	Monoclonal Ab	CLL
	Obinutuzumab [35]	Monoclonal Ab	CLL
CD22	Epratuzumab [37]	Monoclonal Ab	ALL (*orphan drug*)
	Inotuzumab ozogamicin (InO) [38]	Monoclonal Ab	ALL
CD52	Alemtuzumab [41]	Monoclonal Ab	CLL

**Table 2:** Most noteworthy differences between CAR-T based treatments and CAR-NK based treatments.

CD19 CAR-T	CD19 CAR-NK
>400 clinical trials	13 clinical trials
3 commercial products [40]	0 commercial products
Autologous treatment [81]	Allogenic treatment [83]
Poor expansion directly correlated with patient relapse [80]	Short lifespan [78]
Less resistant to genetic engineering	More resistant to genetic engineering
(Up to 80% transduced cells) [81]	(40–60% transduced cells) [82,83]
Activated through CD3ζ 4-1BB and CD28 [88]	Activated through CD3ζ, 4-1BB, DAP10, DAP12, and FcRγ [89,90]
